# Tying Food Addiction to Uncontrolled Eating: The Roles of Eating-Related Thoughts and Emotional Eating

**DOI:** 10.3390/nu17030369

**Published:** 2025-01-21

**Authors:** Alessandro Alberto Rossi

**Affiliations:** 1 Department of Philosophy, Sociology, Education, and Applied Psychology, Section of Applied Psychology, University of Padova, 35131 Padova, Italy; a.rossi@unipd.it; 2 Center for Intervention and Research on Family Studies—CIRF, Department of Philosophy, Sociology, Education, and Applied Psychology, Section of Applied Psychology, University of Padova, 35131 Padova, Italy

**Keywords:** food addiction, binge eating, uncontrolled eating, emotional eating, craving, obesity

## Abstract

**Background**. Food addiction is often linked to overeating and difficulty in controlling eating habits. At the same time, food addiction is often associated with intense eating-related thoughts and emotional eating behaviors. However, despite extensive research on food addiction, the psychological processes that contribute to these outcomes have not been fully examined. Consequently, this study aims to fill that gap by investigating the influence of eating-related thoughts, as well as emotional eating behaviors that may precede episodes of uncontrolled eating. **Methods**. A cross-sectional design was used. A sample of 467 individuals was enrolled from the general population. Participants completed a battery of self-report questionnaires. A sequential mediation analysis with latent variables (i.e., structural equation modeling; SEM) using 5000 bootstrap samples and observed variables was performed. **Results**. The proposed model provides good fit indices. Indeed, food addiction predicts uncontrolled eating behaviors through eating-related thoughts (*p* < 0.001), which were also significantly associated with the emotion-driven eating patterns (*p* < 0.001), revealing a fully mediated model explaining 61.6% of the outcome variance (*R*^2^ = 0.616). **Discussion**. The findings underscore the critical influence of cognitive factors (i.e., eating-related thoughts) in driving maladaptive coping mechanisms like emotional eating. Moreover, emotional eating may act as a precursor to behaviors associated with overeating, which are often rooted in food addiction. **Conclusions**. Recognizing the central role of thoughts and emotions can help clinicians develop more targeted psychological interventions for those experiencing food addiction symptoms.

## 1. Introduction

Obesity has become a global epidemic and is increasingly prevalent in the developed world, with estimates that more than 85% of adults will be diagnosed as being overweight/obese when using the correct criteria for overweight and obesity over the next few decades [1,2]. Obesity is a chronic clinical condition influenced by medical, psychological, and environmental factors that contribute to its development and maintenance [3,4]. One potentially crucial aspect implicated in its onset and persistence is the idea that certain foods have addictive properties [5,6], especially those high in sugar, fat, and salt [7,8,9]. The inherent addictive properties of these food products—namely, highly processed food (HPF) [7,8,10]—which are palatable and (thus) psychologically rewarding, may elicit cravings and withdrawal-like symptoms that may trigger and maintain a dependency response in some individuals [10,11,12,13,14], namely, food addiction (FA).

FA is a complex construct that can be conceptualized as a set of characteristics related to eating disorders (EDs) and substance use disorders (SUDs) [15,16,17,18] which mutually reinforce each other in a vicious cycle, leading the individual to become addicted to food. It should be noted that FA can manifest in several psychological conditions related to EDs [19,20], such as anorexia nervosa (AN), bulimia nervosa (BN), and binge eating disorder (BED), which involves recurrent episodes of uncontrollable eating, often resulting in discomfort or distress [21,22]. In fact, one of the most iconic characteristics of FA is its association with behavioral manifestations related to food consumption, which can range from constant nibbling behaviors (grazing) [23] to binge eating episodes and compulsive eating [24,25]. It thus emerges that FA primarily—although not exclusively [23,26,27]—manifests through uncontrolled food consumption.

These manifestations are often driven and sustained by extremely complex psychological components that are associated with SUDs. Indeed, besides that, HPFs can stimulate neural reward pathways [28,29]; the regular consumption of these foods can result in the development of tolerance, meaning that individuals may need to consume larger quantities of substance (i.e., food) to experience the same level of satisfaction [30]. Also, when the consumption of these HPFs goes unmet, withdrawal symptoms may emerge [31,32], prompting individuals to pursue the same sensations of pleasure and well-being [19,23]. This compulsive urge to consume HPFs may assume the shape of craving symptoms.

As a result, while individuals grapple with FA-related symptoms—such as tolerance, withdrawal, and cravings—these can lead to the development of (intrusive) thoughts specifically tied to food consumption, which influence their behavior, often leading to excessive food intake (e.g., grazing, overeating, and binge eating patterns) [30,33,34]. In fact, symptoms like withdrawal or craving often lead individuals to spend significant amounts of time thinking about the substance, such as HPFs, and these persistent eating-related thoughts may trigger uncontrolled eating behaviors [15,34,35]. Consequently, FA may lead to uncontrolled eating behaviors in response to (e.g., mediated by) these eating-related thoughts and mental states.

Furthermore, as a result of these FA-triggered thoughts, individuals may experience other kinds of negative feelings such as anger, anxiety, preoccupations, worries, and feelings of lack of control that may lead to compulsive overeating and binge/uncontrolled eating behaviors [15,36,37]. Consequently, the urgency to cope with these negative affective states could lead individuals to feel an ‘overwhelming impulse to eat’ [36,38], namely, emotional eating. Indeed, individuals with FA seem to use emotional eating as a coping strategy for psychological distress [36,39,40], suggesting that FA may lead to emotional eating behaviors.

Additionally, this dysfunctional coping strategy for emotion regulation does not seem to fully inhibit the negative and/or distressing emotions for the individual [41,42,43]. As a result, the individual often resorts to more intense and extreme solutions, leading to binge eating behaviors [43,44,45,46,47].

Summarizing, it appears that developing or experiencing symptoms related to food addiction first leads the individual to have thoughts about food consumption [23,30]. These thoughts elicit emotional responses that, in turn, drive emotional eating behaviors as a way to manage them [40,41]. However, even this coping strategy (i.e., dysfunctional coping strategy) appears insufficient to stop the negative and distressing thoughts and emotions, eventually resulting in more intense behaviors such as uncontrolled eating [46,47].

However, to date, although the scientific literature suggests a link between FA symptoms and uncontrolled eating behavior, the path described above does not yet appear to have been fully studied. Consequently, the purpose of the present study was to examine the mediating effect of eating-related thoughts and emotional eating on the relationship between symptoms of FA and uncontrolled eating in a community sample. Indeed, although the link between the previously described variables appears to be fully coherent and dynamically reasonable, it does not seem to have received a clear empirical validation yet; therefore, the objective of this study is to clearly test these connections.

Based on the above-mentioned scientific literature, it was expected that individuals who recall more symptoms of FA would report higher eating-related thoughts, which lead to greater emotional eating and to greater uncontrolled eating. Explicit hypotheses about each path (relationship) between variables were formulated as follows:

**H1.**  *Symptoms of FA, eating-related thoughts, emotional eating, and uncontrolled eating are positively associated with each other;*

**H2.**  *Symptoms of FA predict uncontrolled eating via eating-related thoughts and emotional eating;*

**H3.**  *Symptoms of FA directly predict uncontrolled eating.*

In other words, it was hypothesized that the positive associations between symptoms of FA on uncontrolled eating behaviors would be partially mediated by the presence of binge eating thoughts and emotional eating.

## 2. Methods and Materials

### 2.1. Procedure

According to previous studies, the snowball sampling technique [48] was used to recruit participants from the general population via social media platforms such as Facebook, Twitter/X, or Instagram [49]. To begin, participants were required to click a link that led to a Qualtrics survey. The recruitment materials provided comprehensive information regarding eligibility requirements and other relevant details to help participants make informed decisions, including guarantees of anonymity for their responses. Participants who consented to take part completed the survey online. It was specified that no financial compensation was offered for completing the survey; therefore, only voluntary participants were enrolled.

The criteria for inclusion were as follows: (A) participants should be 18 years or older; (B) they should be native Italian speakers; (C) they need to provide complete responses; and (D) they must sign an online informed consent form. This study received approval from the Ethics Committee of the University of Padua (protocol n° 3558).

### 2.2. Sample Size Determination

The sample size was predetermined based on the primary statistical analysis, specifically using the “*n:q* criterion”, which refers to the ratio of participants (*n*) to model parameters (*q*) [50]. According to established guidelines, a minimum of 10 participants per parameter was required to ensure adequate statistical power for the proposed model. Given that the model tested had 45 parameters, this necessitated a minimum enrollment of 450 participants to meet this criterion.

### 2.3. Participants

A sample of 551 participants started the survey; however, 84 of them did not complete it, and were therefore excluded (inclusion criterion C). A total of 467 participants were recruited. The sample comprised 125 males (26.8%) and 342 females (73.2%), with ages ranging from 18 to 71 years (*mean* = 31.25, *SD* = 13.52) and BMIs ranging from 15.62 to 40.66 (*mean* = 23.92; *SD* = 5.38). Additional details can be found in Table 1.

### 2.4. Measures

The demographic information gathered included age, gender, education level, marital status, and employment status. Additionally, participants were requested to self-report their height and weight to facilitate the calculation of their Body Mass Index (BMI), as well as to indicate the presence of a diagnosis of an eating disorder [51].

#### 2.4.1. The Modified Yale Food Addiction Scale 2 (mYFAS 2)

The mYFAS 2.0 [52] is a self-assessment instrument aimed at assessing the frequency of food addiction symptoms within the last year. It is composed of 13 items rated on an 8-point Likert-type scale. Among these items, 11 align with the DSM-5 criteria for substance use disorder (SUD), such as tolerance, withdrawal, and craving. The remaining two focus on food-related psychological and emotional distress experienced by the individual. To establish a diagnosis of food addiction (FA), two scoring methods are utilized: the symptom count score, which tallies the number of diagnostic criteria met, and the diagnostic score, which evaluates the degree of impairment or emotional distress, categorizing it as No FA, mild FA, moderate FA, or severe FA. In the present study, the symptom count score was used, which higher scores indicate a greater amount of symptoms endorsed. In this study, the Italian version of the mYFAS 2.0 was used [53], showing good internal consistency values with a McDonald’s omega for categorical data of 0.832.

#### 2.4.2. The Binge Eating Scale (BES)

The BES [35] is a self-report tool designed to assess the severity of feelings, cognitions, and behaviors related to binge eating. It consists of 16 items that capture the core characteristics of binge eating disorder (BED), in which each item offers from three to four levels of symptom descriptions, and higher scores indicate higher severity levels. The BES is composed of two distinct scales: Feelings/Cognitions (F/C), and Behaviors (B). The B scale strictly evaluates binge eating behaviors such as consuming large amounts of food quickly. Opposite to this, the F/C scale evaluates thoughts, feelings, and cognitions that precede eating behaviors (not strictly limited to binge eating). In this study, the Italian version of the BES was used [54]. Although the semantic content of the two scales is different, given the potential strong correlation between them [55], only the F/C scale—assessing thoughts, feelings, and cognitions related to binge eating—was used for the main analysis in this study. In this research, the internal consistency measured by McDonald’s omega was found to be 0.801.

#### 2.4.3. The Three-Factor Eating Questionnaire—Revised—18 (TFEQ-R-18)

The TFEQ-R-18 [56] is a well-established and psychometrically robust questionnaire designed to evaluate three primary cognitive and behavioral aspects related to eating disorders: Cognitive Restraint (CR), Uncontrolled Eating (UE), And Emotional Eating (EE). It comprise 18 items rated on a 4-point Likert-type scale, and higher scores indicate greater levels in each of these areas. The CR dimension assesses individuals’ attempts and concerns regarding the regulation of their food consumption to manage body weight and shape. The EE dimension refers to the inclination to eat in reaction to emotional triggers, whether they are positive or negative, such as feelings of distress or other negative emotions. Meanwhile, the UE dimension evaluates the propensity to lose self-control and engage in overeating. Given the semantic content of the items of the CR scale are not of interest for the present study, it was not included in the statistical analyses. In this study, the Italian version of the TFEQ-R-18 was used [57]. In this research, the internal consistency, measured by McDonald’s omega, was found to be 0.864 for the EE scale and 0.877 for the UE scale.

### 2.5. Statistical Analysis

**Figure 1:**
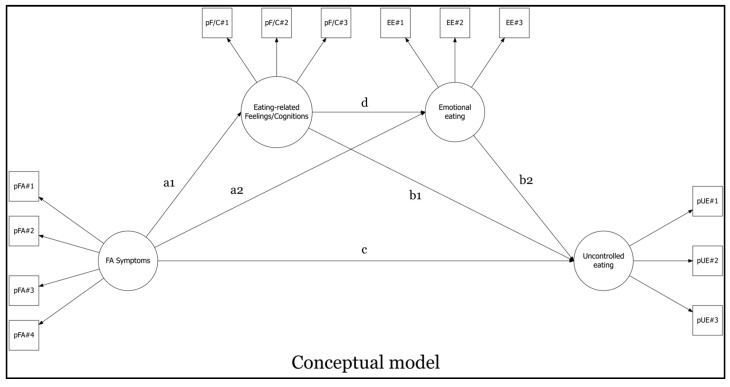
Structural equation model—conceptual representation.

Statistical analyses were performed using the software R (v. 4.3.2). In line with the inclusion and exclusion criteria, all observations were complete and contained no missing data. To meet the goals of this study, different phases were conducted [58,59,60,61]. (*i*) Prior to executing the main statistics, preliminary analyses were undertaken to evaluate the strength of the relationships among the observed variables [62,63]. Also, in line with previous studies, a multivariate multiple regression was conducted to explore the presence of potential socio-demographic factors that might influence the relationship between the variables. (*ii*) A confirmatory factor analysis (CFA; Diagonally Weighted Least Squares estimator, DWLS) was used to assess the measurement model of each questionnaire by applying traditional goodness-of-fit indices (χ^2^, RMSEA, CFI, and SRMR) and adhering to the recommended cutoff values: (A) a non-statistically significant χ^2^, (B) an RMSEA of less than 0.08, (C) a CFI greater than 0.95, and (D) an SRMR below 0.08 [50,64,65]. (*iii*) An evaluation of common method bias was conducted [63,66,67,68]. (*iv*) A structural equation model with latent variables (SEM) was specified: FA symptoms (X) were regressed on Uncontrolled Eating (Y) through binge eating thoughts (M1) and Emotional Eating (M2) (see Figure 1) [69,70,71,72]. (*v*) Latent factors were operationalized through the use of parcels as indicators [73,74]. Specifically, a partially disaggregated item–parcel method was employed [75,76], using a strategy focused on maintaining a balance between items and constructs (i.e., item-to-construct balance strategy) [73,77]. It should be noted that, since the EE scale of the TFEQ-18-R consists of only 3 items, it is not possible to create parcels due to the low number of items. Therefore, for the 3 items composing the EE scale, a fully disaggregated approach was used [68,73,74]. (*vi*) The SEM using parcels as indicators was conducted employing the maximum likelihood (ML) estimator along with a bootstrap resampling procedure of 5000 iterations using the Bollen–Stine method to address the non-ideal normality of the parcels. Model fit was evaluated using the χ^2^ test, RMSEA, CFI, and SRMR, in accordance with their recommended cutoff values [50,64]. All regression coefficients presented in the Results section were reported as unstandardized (β).

## 3. Results

### 3.1. Preliminary Analysis

Correlation analyses (H1) revealed a moderate to strong positive relationship among the psychological variables involved in the SEM. However, none of these factors appear to exceed critical thresholds. Similarly, for the socio-demographic variables, none seem to show markedly strong associations with the variables of interest (Table 2 and Table S1 in Supplementary Materials).

### 3.2. Structural Models

The mYFAS2-0 scale showed adequate goodness-of-fit indices: χ^2^ (44) = 37.368; *p* = 0.750; RMSEA = 0.000; 90%CI 0.000–0.023; *p*(RMSEA < 0.05) = 1, CFI = 1, SRMR = 0.074. Also, the BES F/C subscale showed adequate fit indices: χ^2^ (14) = 16.837; *p* = 0.265; RMSEA = 0.021; 90%CI 0.000–0.52; *p*(RMSEA < 0.05) = 0.938, CFI = 0.999, SRMR = 0.042. Lastly, the TFEQ-R-18 (‘Emotional Eating’ and ‘Uncontrolled Eating’ subscales) showed adequate goodness-of-fit indices: χ^2^ (53) = 155.534; *p* < 0.001; RMSEA = 0.064; 90%CI 0.053–0.076; *p* (RMSEA < 0.05) = 0.021, CFI = 0.991, SRMR = 0.053.

### 3.3. Harman’s Single-Factor Test

Harman’s single-factor test showed an absence of the ‘common method bias’. Indeed, the CFA with four correlated factors provided adequate fit indices: χ^2^ (399) = 805.206; *p* < 0.001; RMSEA = 0.047; 90%CI 0.042–0.051; *p*(RMSEA < 0.05) = 0.874, CFI = 0.988, SRMR = 0.078. Opposite, the single-factor CFA provided poor-fit indices: χ^2^ (405) = 1722.523; *p* < 0.001; RMSEA = 0.084; 90%CI 0.080–0.088; *p*(RMSEA < 0.05) < 0.001, CFI = 0.960, SRMR = 0.111. The model comparison further suggested the absence of the ‘common method bias’: Δχ^2^ (6) = 917.32, *p* < 0.001; |ΔRMSEA| = 0.037, and |ΔCFI| = 0.028.

### 3.4. Latent Mediation Model with Parcels

Item parcels were almost normally distributed, and they were statistically significantly associated with the latent variable and exhibited standardized factor loading higher than 0.5, ranging from 0.628 to 0.885 (see Table S2; Supplementary Materials).

**Figure 2:**
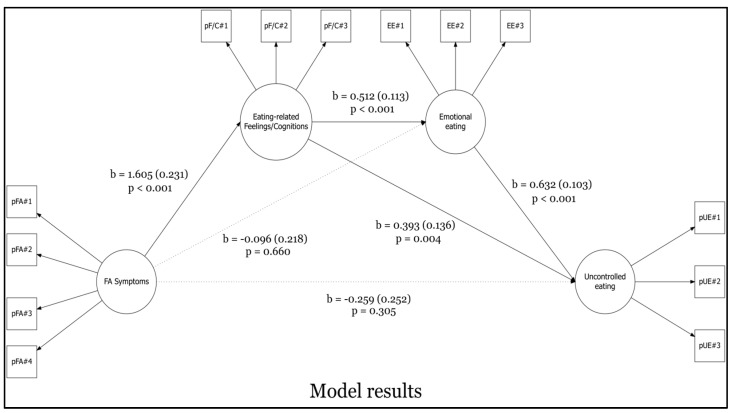
Structural equation model with unstandardized regression coefficients, standard errors, and associated *p*-values.

The model (see Figure 1) provided adequate goodness-of-fit indices: χ^2^ (59) = 132.909; *p* < 0.001; RMSEA = 0.052; 90%CI 0.040–0.064; *p*(RMSEA < 0.05) = 0.384 *ns*, CFI = 0.978, SRMR = 0.035. Results are reported in Figure 2 and Table 3.

The hypothesized model (H2) showed that FA symptoms (X) were positively associated with Binge Eating Feelings/Cognitions (M1), *path a*1: β = 1.605 (SE = 0.231) [95%CI: 1.241; 2.154], z = 6.935, *p* < 0.001. At the same time, Binge Eating Feelings/Cognitions (M1) were positively associated with Emotional Eating (M2), *path d*: β = 0.512 (SE = 0.113) [95%CI: 0.314; 0.752], z = 4.534, *p* < 0.001. Also, Emotional Eating (M2) predicted Uncontrolled Eating behaviors (Y), *path b*2: β = 0.632 (SE = 0.103) [95%CI: 0.438; 0.844], z = 6.121, *p* < 0.001. Lastly, (H3) FA symptoms (X) were no longer associated with Uncontrolled Eating behaviors (Y), *path c’*: β = −0.259 (SE = 0.252) [95%CI: −0.868; 0.098], z = −1.026, *p* = 0.305, thus suggesting a totally mediated path.

Considering the other paths, interestingly, FA symptoms (X) were no longer associated with emotional eating (M2), *path a*2: β = −0.096 (SE = 0.218) [95%CI: −0.585; 0.264], z = −0.440, *p* = 0.660 *ns*. Opposite, in line with expectations, Binge Eating Feelings/Cognitions (M1) predicted Uncontrolled Eating behaviors (Y), *path b*1: β = 0.393 (SE = 0.136) [95%CI: 0.174; 0.712], z = 2.892, *p* = 0.004.

Moreover, an examination of the indirect path was conducted. The total indirect effect (FA symptoms → Binge Eating Feelings/Cognitions → Emotional Eating → Uncontrolled Eating behaviors) was statistically significant: β = 0.519 (SE = 0.130) [95%CI: 0.312; 0.827], z = 3.986, *p* < 0.001. Also, the total model effect was inspected, the results being statistically significant: β = 0.831 (SE = 0.116) [95%CI: 0.635; 1.083], z = 7.193, *p* < 0.001. The degree of explained variance (*R*^2^) was equal to 61.6% (*R*^2^ = 0.616).

## 4. Discussion

Over the years, numerous studies have highlighted the significant impact of FA on the physical and mental health of individuals. Consequently, the present research aimed to provide a deeper understanding of the association between FA symptoms and dysfunctional eating behaviors such as uncontrolled eating in order to design timely psychological interventions and to explore their effects on other constructs within research contexts.

Indeed, there has been a steady increase in the prevalence of obesity and EDs worldwide, particularly in industrialized countries where expenditure for medical and psychological conditions related to HPF consumption is markedly high [78,79]. The literature acknowledges that these foods [6,7,10]—highly palatable and psychologically rewarding—can lead some individuals to develop an addiction to food, known as FA [80]. FA appears to play a pivotal role in this epidemic, contributing to unhealthy eating behaviors such as grazing, binge eating, or uncontrolled eating, which, over time, promote the development of obesity [23,81].

This study is the first to enhance the understanding of how eating-related thoughts, along with emotional eating, contribute to the development of disordered eating behaviors starting from FA symptoms—examining the conceptual model illustrated in Figure 1 and Figure 2—in a sample of individuals enrolled from the general population. This research explores the relationship between (A) FA symptoms, (B) thoughts, (C) emotion-driven behaviors, and (D) uncontrolled eating.

The primary findings of this research indicate that FA symptoms do not have a direct effect on uncontrolled eating, as their relationship is fully mediated by binge eating thoughts and emotional eating, accounting for a notable portion of the variance in uncontrolled eating. This highlights the critical role of cognitive and emotional components in mediating the effects of FA symptoms on the tendency to engage in uncontrolled eating behaviors [24,34,82]. The mediating influence of these factors suggests that uncontrolled eating behaviors may be triggered by the development of food-focused thought processes in individuals exhibiting FA symptoms [34]. Indeed, FA includes symptoms such as withdrawal and craving for HPFs. The presence of these elements leads individuals to experience persistent and often intrusive thoughts, consuming much of their time [30]. These thoughts frequently orbit around food, concerns about it, and strategies for obtaining, consuming, and savoring it, despite awareness of the harm HPFs may cause. The prominence of food and eating-related worries, fears, desires, and cravings underscores the significant impact of dysfunctional eating-related thoughts, which are critical variables in various disordered eating behaviors and eating disorders [44,82,83]. At the same time, these thoughts contribute to the development of negative emotions. These emotions often include anxiety, anger, and frustration, stemming from both the condition of dependency and the inability to immediately satisfy the need to consume the desired substance [41,43]. Additionally, feelings of worry and fears of losing control are frequently present. Such emotions are often overwhelming, prompting the individual to employ emotional eating (e.g., a coping strategy) to manage them [42]. Finally, the model shows that emotional eating—preceded by FA symptoms and eating-related thoughts—drives individuals to engage in disordered eating behaviors that may lead to compulsive overeating, uncontrolled eating, and a loss of control over food consumption in a maladaptive effort to cope, further reinforcing and maintaining the cycle of addiction [49,84,85,86,87,88].

These findings suggest that FA symptoms and uncontrolled eating behaviors are not necessarily linearly connected, but that their association is totally mediated by thoughts and emotion-driven behaviors, with particular emphasis on the former. Indeed, it is important to highlight that, when controlling for eating-related thoughts, FA symptoms are no longer associated with either emotional eating or uncontrolled eating. This result highlights the crucial role of thoughts as a key element in developing coping strategies and dysfunctional eating behaviors [34]. On the one hand, these findings suggest that emotional eating must also rely on a cognitive component to manifest and that, consistent with the literature, emotions seem to follow the individual’s mental state. On the other hand, these results appear to indicate that symptoms such as withdrawal or craving, once the effect of thoughts is controlled for, do not seem capable of leading to maladaptive behaviors.

It is important to emphasize that, in this model, thoughts (related to food) were placed sequentially before emotion-driven behaviors. It is crucial to note that the relationship between emotions and thoughts has been the subject of numerous studies, revealing that they can influence each other [89,90]. Indeed, some theories suggest that thoughts often precede emotions, while others propose the opposite [90]. Indeed, there are theories that postulate emotional states as antecedents of thoughts, suggesting that emotions can manifest before conscious thoughts [91,92,93,94]. For example, physiological responses to stimuli can rapidly trigger emotions, often before an individual has the time to process or articulate their thoughts about the situation. This perspective is supported by studies indicating that emotional reactions are fundamental in decision making and cognitive processes, as individuals deprived of emotional responses often encounter difficulties in logical reasoning and decision making [91]. However, this is not the case for the variables involved in this research. The hypothesis described above might be valid in specific conditions, such as individuals experiencing severe food deprivation or intense craving states [34,85]. In fact, the participants evaluated in this study did not exhibit such characteristics. Instead, this study assessed the number of FA symptoms over the past year [52], being, therefore, in a chronic rather than an acute condition. Based on this chronicity of symptoms, a significant body of empirical evidence highlights the role of cognitive appraisal in shaping emotional experiences, leading to the formulation of feelings based on the cognitive interpretation of the situation [95,96,97]. According to these theories, thoughts can (also) influence how we experience and define emotions [95,97]. Thus, the relationship between emotions and thoughts is dynamic and context-dependent, suggesting an interconnectedness between the two. For example, some research indicates that children’s understanding of how thoughts influence emotions develops over time [89]. This suggests that, while emotions may arise first, conscious thought can later modify or influence these emotional states. This interaction underscores that both processes are essential in shaping human behavior and experiences [89,98]. Lastly, regarding the variables measured in this study, it is worth emphasizing once again that the variable ‘Emotional Eating’ is tied to a behavioral actions influenced by emotions rather than to simple/pure emotional states [41,42]. Therefore, the reasoning behind positioning these variables (thoughts and emotions related to food) in this way appears more plausible [99]: the FA symptoms from the past year lead to eating-related thoughts and emotional eating behaviors.

In summary, these findings enhance our understanding of eating-related thoughts and emotional-driven behaviors among individuals exhibiting core features of disordered eating behaviors [49,88]. Additionally, they align with existing research indicating that thoughts about food significantly contribute to the variance observed in uncontrolled eating. Specifically, studies have shown that individuals with eating-related craving symptoms demonstrate higher levels of overwhelming eating-related thoughts. Also, studies have shown that these thoughts may trigger emotional eating behaviors, which in turn lead to binge eating. However, prior studies did not specifically examine the relationship between these variables together starting from FA symptoms. This study provides new insights into the nature of FA symptoms by elucidating the mechanisms connecting eating-related thoughts and emotional eating to maladaptive patterns of eating behaviors. However, it is important to note that these findings are based on a moderately large sample recruited from the general population, and further studies on clinical samples are needed to provide additional confirmation.

Thus, the shift from FA to thoughts of uncontrolled eating represents a complex interplay between psychological, physiological, emotional, and behavioral elements. Increasingly, FA has been considered a major marker for a variety of eating disorders, mainly BED, characterized by compulsive eating behaviors and loss of control over eating despite adverse consequences. However, this association is fully mediated by psychological factors. Individuals with FA symptoms frequently report persistent thoughts of concern about food; thoughts about its availability, preparation, and anticipatory ideas related to its consumption leading to emotional eating. The incidence of emotional eating can further cycle FA and binge eating because temporary relief may be obtained through food consumption, but will ultimately result in negative psychological effects that continue to support disordered eating [49,84,85,86,87,88].

### 4.1. Strengths and Limitations

This research has several limitations. Firstly, it relies on self-report measures without clinical interviews or behavioral measures to properly evaluate the severity of FA symptoms as well as uncontrolled eating behaviors. Nonetheless, all assessment tools used for this study are well validated and consistently measure the target construct. Secondly, due to the cross-sectional design, causal relationships cannot be established; however, robust statistical methods enabled the testing of hypotheses regarding associations and influences among constructs. Future studies could employ longitudinal designs to explore the long-term effects of FA symptoms [100]. Thirdly, this study examined a limited set of variables, and other confounding factors—such as anxiety, impulsivity, and emotion regulation difficulties—may also play a role, warranting investigation in future research. Fourthly, the sample was slightly skewed towards females, suggesting that future studies should consider gender and ethnic differences. Furthermore, it is important to note that although the sample in the present study is moderately large, the results are only extendable to individuals drawn from the general Italian population, making them susceptible to potential representation bias. For this reason, future studies could aim to extend the sample—making it more representative of the Italian population—and/or test the same hypotheses in individuals with different clinical conditions, such as eating and feeding disorders and/or obesity. Lastly, it should be noted that since all the scales used are related to the domain of excessive food intake and overeating, there might be semantic redundancy among the items of the scales, potentially amplifying the correlations between the scales themselves. However, it is important to note that the correlations among the scales used do not exceed the critical threshold of 0.84 [62,83] and are all associated with different instruments. In this regard, the only potentially problematic correlations would be those between scales within the same instrument—in this case, the TFEQ-R-18. However, the correlation between the EE scale and the UE scale is 0.654, which is well below the critical threshold. For these reasons, there is no excessive concern, and the results can be considered sufficiently reliable.

Despite these limitations, this research has notable strengths. Firstly, it involved a large and diverse sample of individuals. Secondly, this study employed rigorous methodology and statistical analyses based on validated procedures. Third, this research also contributes to a deeply understanding of the relationships among the FA symptoms, binge eating-related thoughts, emotional eating, and uncontrolled eating. Additionally, it establishes a connection between some of the most important psychological variables upon which modern therapies are based, namely the centrality of thoughts and emotions in behavioral processes.

### 4.2. Implications for Clinical and Research Practice

The clinical implications of this study are significant for both research and clinical practice. Regarding the former, this study provides a solid experimental foundation to enhance our understanding of one of the most important and debated phenomena in recent years.

At the same time, the clinical applications align closely with what was outlined in the previous section. Indeed, it is worth emphasizing that modern evidence-based psychotherapeutic approaches aim to improve the management of (dysfunctional) thoughts, metacognitions, intrusive thoughts, and negative emotions [101,102,103,104,105]. This study highlights the key and central role of thoughts and emotions in behavioral processes related to uncontrolled eating, thus offering an initial guideline for clinicians working with individuals exhibiting FA symptoms [106].

## 5. Conclusions

This study contributes to the understanding of disordered eating behaviors associated with FA and its possible treatment. The findings indicate that both eating-related thoughts and emotional eating serve as mediators in the relationship between FA symptoms and uncontrolled eating. These results underscore the significant role that thoughts and emotions play in the development and persistence of dysfunctional eating behaviors.

## Figures and Tables

**Table 1:** Sample’s descriptive statistics.

Variable	Descriptive Statistics	
Age (*M*. *SD*)	31.25	13.52
Sex (*n*. %)		
Male	125	26.8%
Female	342	73.2%
Marital status (*n*. %)		
Single	182	39.0%
In a relationship	184	39.4%
Married	75	16.1%
Separated/divorced	23	4.9%
Widowed	3	0.6%
Education (*n*. %)		
Middle school	33	7.1%
High school	224	48.0%
University	188	40.3%
Ph.D.	22	4.7%
Work status (*n*. %)		
Student	225	48.2%
Full-time worker	152	32.5%
Part-time worker	18	3.9%
Entrepreneur	48	10.3%
Housewife	3	0.6%
Unemployed	15	3.2%
Retired	6	1.3%
BMI Class (*n*, %)		
Severely underweight (<16)	2	0.4%
Underweight (16–18.49)	49	10.5%
Normal weight (18.5–24.99)	269	57.6%
Overweight (25–29.44)	74	15.8%
Class I obesity (30–34.99)	58	12.4%
Class II obesity (35–39.99)	12	2.6%
Class III obesity (>40)	3	0.6%
Eating disorder (*n*. %)		
No eating disorder	401	85.9%
Anorexia Nervosa	21	4.5%
Bulimia Nervosa	14	3.0%
Binge eating disorder	30	6.4%
Eating disorder not otherwise specified	1	0.2%

Note: *N* = 467. M = mean; SD = standard deviation.

**Table 2:** Study 1. Descriptive statistics and correlations among variables.

		Descriptive Statistics				Correlations					
		M	SD	SK	K	1	2	3	4	5	6
1	mYFAS2 Symptom Count	1.00	1.89	2.56	7.03	-					
2	BES total score	8.51	7.36	1.27	1.77	0.712	-				
3	BES Feelings/Cognitions	3.97	3.82	1.27	1.42	0.689	0.919	-			
4	BES Behaviors	4.55	4.13	1.30	1.92	0.631	0.931	0.713	-		
5	TFEQ-R-18 Emotional eating	6.63	2.63	0.34	−0.82	0.474	0.608	0.559	0.567	-	
6	TFEQ-R-18 Uncontrolled Eating	17.49	5.62	0.77	0.23	0.460	0.651	0.568	0.634	0.654	-

Note: all correlations are statistically significant with *p* < 0.001. M = mean, SD = standard deviation, SK = skewness, K = kurtosis.

**Table 3:** Summary of standardized parameter estimates (Beta) with 95% confidence intervals of the tested model (Figure 2).

		β*	β (SE)	95%CI [L - U]	z-Value	*p*-Value	*R* ^2^
FA symptoms (X) → Feelings/Cognitions (M1)	a1	0.849	1.605 (0.231)	[1.241; 2.154]	6.935	*p* < 0.001	0.720
Feelings/Cognitions (M1) → Emotional Eating (M2)	d	0.724	0.512 (0.113)	[0.314; 0.752]	4.534	*p* < 0.001	0.441
Emotional Eating (M2) → Uncontrolled Eating (Y)	b2	0.524	0.632 (0.103)	[0.438; 0.844]	6.121	*p* < 0.001	0.616
FA symptoms (X) → Uncontrolled Eating (Y)	c’	−0.160	−0.259 (0.252)	[−0.868; 0.098]	−1.026	0.305	
FA symptoms (X) → Emotional Eating (M2)	a2	−0.072	−0.096 (0.218)	[−0.585; 0.264]	−0.440	0.660	
Feelings/Cognitions (M1) → Uncontrolled Eating (Y)	b1	0.461	0.393 (0.136)	[0.174; 0.712]	2.892	0.004	
Indirect effect of X on Y via M1	a1*b1	0.391	0.631 (0.286)	[0.256; 1.340]	2.209	0.027	
Indirect effect of X on Y via M2	a2*b2	−0.038	−0.061 (0.132)	[−0.346; 0.178]	−0.458	0.647	
Indirect effect of X on Y via M1 and M2	a1*d*b2	0.322	0.519 (0.130)	[0.312; 0.827]	3.986	*p* < 0.001	
Total effect of X on Y		0.515	0.831 (0.116)	[0.635; 1.083]	7.193	*p* < 0.001	

Note: β* = standardized beta; β = unstandardized beta; SE = standard error; 95%CI = 95% confidence intervals (lower/upper) for the unstandardized beta; *R*^2^ = explained variance; *p*(…) = item parcel; FA symptoms = symptom count of the mYFAS 2; Feelings/Cognitions = Binge Eating Feelings/Cognitions subscale of BES; Emotional Eating = Emotional Eating scale of the TFEQ-18-R; Uncontrolled Eating = Uncontrolled Eating scale of the TFEQ-18-R.

## Data Availability

Data are available on a reasonable request due to privacy and ethical restrictions.

## Supplementary Materials

The following supporting information can be downloaded at: https://www.mdpi.com/article/10.3390/nu17030369/s1, Table S1: Multivariate multiple regression between socio-demographic variables and variables used in the SEM model; Table S2: Item parcels’ descriptive statistics and factor loadings (λ).
