# Supplementary material for: Tying Food Addiction to Uncontrolled Eating: The Roles of Eating-Related Thoughts and Emotional Eating
Source: Nutrients. 2025 Jan 21;17(3):369. doi: 10.3390/nu17030369 (PMC11819927; doi:10.3390/nu17030369)
Supplement: Supplementary file 1 [file nutrients-17-00369-s001.zip › nutrients-3437979-supplementary.pdf]

Tying Food Addiction to Uncontrolled Eating: The Roles of Eating-Related Thoughts and Emotional Eating

Alessandro Alberto Rossi[1,2]

[1] *Department of Philosophy, Sociology, Education, and Applied Psychology, Section of Applied Psychology, University of Padova, Padova, Italy.*

[2] *Interdepartmental Center for Family Research, University of Padova, Padova, Italy.*

SUPPLEMENTARY MATERIAL

CORRESPONDING AUTHOR:

Alessandro Alberto Rossi

Department of Philosophy, Sociology, Education, and Applied Psychology, University of Padova, Padova, Italy

Email: a.rossi@unipd.it

Table S1. Multivariate multiple regression between socio-demographic variables and variables used in the SEM model.

		β*	β (SE)	95%CI [L - U]	z-value	*p*-value
Outcome	FA symptoms					
Predictor	Age	0.098	0.014(0.012)	[-0.009; 0.036]	1.189	0.234
Predictor	Education	0.074	0.202(0.121)	[-0.034; 0.439]	1.675	0.094
Predictor	Work status	-0.082	-0.115(0.054)	[-0.220; -0.009]	-2.135	0.033
Predictor	Civil status	0.061	0.129(0.146)	[-0.158; 0.416]	0.879	0.379
Predictor	Sex	0.154	0.658(0.166)	[0.332; 0.983]	3.961	< 0.001
Predictor	BMI Class	0.277	0.536(0.112)	[0.316; 0.756]	4.775	< 0.001
Outcome	Feelings/Cognitions					
Predictor	Age	-0.061	-0.017(0.023)	[-0.062; 0.028]	-0.748	0.454
Predictor	Education	0.047	0.261(0.263)	[-0.254; 0.775]	0.993	0.321
Predictor	Work status	-0.008	-0.021(0.121)	[-0.259; 0.216]	-0.176	0.860
Predictor	Civil status	0.019	0.084(0.272)	[-0.449; 0.616]	0.307	0.758
Predictor	Sex	0.235	2.023(0.346)	[1.345; 2.701]	5.845	< 0.001
Predictor	BMI Class	0.303	1.183(0.254)	[0.686; 1.680]	4.667	< 0.001
Outcome	Emotional eating					
Predictor	Age	-0.134	-0.026(0.016)	[-0.058; 0.006]	-1.583	0.113
Predictor	Education	0.047	0.178(0.173)	[-0.160; 0.517]	1.033	0.302
Predictor	Work status	0.038	0.074(0.088)	[-0.099; 0.247]	0.835	0.404
Predictor	Civil status	0.090	0.266(0.200)	[-0.126; 0.659]	1.330	0.184
Predictor	Sex	0.287	1.702(0.270)	[1.174; 2.230]	6.316	< 0.001
Predictor	BMI Class	0.225	0.606(0.174)	[0.264; 0.947]	3.477	0.001
Outcome	Uncontrolled Eating					
Predictor	Age	-0.269	-0.112(0.035)	[-0.181; -0.043]	-3.181	0.001
Predictor	Education	0.066	0.539(0.384)	[-0.214; 1.292]	1.403	0.161
Predictor	Work status	-0.013	-0.056(0.204)	[-0.456; 0.344]	-0.274	0.784
Predictor	Civil status	0.201	1.269(0.441)	[0.405; 2.133]	2.878	0.004
Predictor	Sex	0.138	1.747(0.578)	[0.613; 2.880]	3.020	0.003
Predictor	BMI Class	0.133	0.763(0.359)	[0.060; 1.467]	2.126	0.033

Note: β* = standardized beta; β = unstandardized beta; se = standard error; 95%CI = 95% confidence intervals (lower/upper) for the unstandardized beta; FA symptoms = Symptom count of the mYFAS 2; Feelings/cognitions = Binge Eating Feelings/Cognitions subscale of BES; Emotional Eating = Emotional Eating scale of the TFEQ-18-R; Uncontrolled Eating = Uncontrolled Eating scale of the TFEQ-18-R.

Table S2. Item parcels' descriptive statistics and factor loadings (λ).

	Descriptive statistics				Factor loadings	
	M	SD	SK	K	λ	R^2
Food addiction symptoms (X)						
pFA#1	0.123	0.232	1.984	3.458	0.837	0.700
pFA#2	0.082	0.197	2.631	6.862	0.813	0.662
pFA#3	0.075	0.190	2.765	7.571	0.693	0.480
pFA#4	0.082	0.225	2.800	7.239	0.628	0.395
Feelings / Cognitions (M1)						
pF/C#1	0.767	0.852	1.068	0.215	0.706	0.498
pF/C#2	0.513	0.634	1.549	2.440	0.737	0.543
pF/C#3	0.469	0.521	1.742	4.093	0.806	0.649
Emotional eating (M2)						
EE#1	0.674	0.586	0.604	-0.319	0.782	0.612
EE#2	0.284	0.466	2.513	7.911	0.883	0.779
EE#3	0.558	0.569	1.305	1.904	0.797	0.635
Uncontrolled Eating (Y)						
pUE#1	2.021	0.672	0.716	0.039	0.856	0.733
pUE#2	1.802	0.697	0.729	-0.056	0.885	0.784
pUE#3	2.006	0.718	0.532	-0.217	0.793	0.629

Note: M = mean, SD = standard deviation SK = Skewness, K = Kurtosis; λ = unstandardized factor loading; se = standard error; λ* = unstandardized factor loading; R^2 = explained variance; p(…) = item parcel; Food addiction symptoms = Symptom count of the mYFAS 2; Feelings/cognitions = Binge Eating Feelings/Cognitions subscale of BES; Emotional Eating = Emotional Eating scale of the TFEQ-18-R; Uncontrolled Eating = Uncontrolled Eating scale of the TFEQ-18-R. As mentioned in the dedicated section, since the EE scale of the TFEQ-18-R consists of only 3 items, it is not possible to create parcels. For this reason, the latent factor was directly constructed using the 3 items that comprise it.
